# Characterization of a novel sn1,3 lipase from *Ricinus communis* L. suitable for production of oleic acid-palmitic acid-glycerol oleate

**DOI:** 10.1038/s41598-021-86305-z

**Published:** 2021-03-25

**Authors:** Yue Li, Guorui Li, Huajun Sun, Yongsheng Chen

**Affiliations:** 1 Department of Food Science, Shenyang Agricultural University, Shenyang, 110866 People’s Republic of China; 2 College of Life Sciences and Food Engineering, Inner Mongolia University for Nationalities, Tongliao, 028000 People’s Republic of China; 3 Inner Mongolia Industrial Engineering Research Center of Universities for Castor, Tongliao, 028000 People’s Republic of China; 4 Inner Mongolia Key Laboratory of Castor Breeding, Tongliao, 028000 People’s Republic of China; 5 Inner Mongolia Collaborate Innovation Center for Castor, Tongliao, 028000 People’s Republic of China; 6 Department of Food Science, Heilongjiang Bayi Agricultural University, Daqing, 163000 People’s Republic of China

**Keywords:** Biochemistry, Biological techniques

## Abstract

The hydrolysis properties of lipase in castor was evaluated using two different substrate forms (tripalmitic glycerides and trioleic glycerides) to catalyze the reaction under different operational conditions. RcLipase was obtained from castor seeds and results show that RcLipase is a conservative serine lipase with a conserved catalytic center (SDH) and a conserved pentapeptide (GXSXG). This enzyme exhibited the greatest activity and tolerance to chloroform and toluene when it was expressed in *Pichia pastoris* GS115 at 40 ℃ and pH 8.0. Zn and Cu ions exerted obvious inhibitory effects on the enzyme, and displayed good hydrolytic activity for long-chain natural and synthetic lipids. HPLC analysis showed that this enzyme has 1,3 regioselectivity when glycerol tripalmitate and oleic acid are used as substrates. The fatty acid composition in the reaction product was 21.3% oleic acid and 79.1% sn-2 palmitic acid.

## Introduction

Triacylglycerols (TAG), an energy storage substance, exist in the seeds of many eukaryotic plants. TAG is hydrolyzed by lipase to produce free fatty acids and corresponding glycerides, in which free fatty acids provide energy and substances through oxidative catabolism for seed growth during seed germination^1^. Lipase is an essential triglyceride hydrolase, mainly involved in the hydrolysis of triacylglycerol, and can also catalyze ester formation, transesterification, alcoholysis, acid hydrolysis, aminolysis, and deacetylation^2^. Lipase has been widely used in many industries, such as food oil production, chemical production, agriculture, paper making, etc., because of its substrate specificity^3^. Lipase is widespread in seeds of oil plants, animal pancreas, other adipose tissue, and fungi and yeasts. Among these, lipases derived from yeasts and fungi are the most important commercial application enzymes, while plant-derived commercial lipases are relatively rare^4^.

The active site of lipase is located at the top of the central beta-sheet in the protein structure, and the main components at the edges of active sites are hydrophobic residues, which interact with hydrophobic bases^5^. The catalytic triad of the lipase catalytic center is very conservative, consisting of nucleophilic serine (Ser), aspartic acid (Asp) or glutamic acid (Glu), and histidine (His), and Ser is usually located after the fifth beta fold and before the alpha helix, while Asp or Glu is normally located after the seventh beta fold, with His after the eighth beta fold^6^. The first residue of the oxygen anion cavity is located at the fifth beta fold near the catalytic active center of the Ser residue at the conservative nucleophilic elbow, and is usually located at the X2 position of G-X1-S-X2-G, while the second residue of the oxygen anion is usually located at the N end of the lipase on the loop ring between the third beta-chain and the alpha-a-helix^7^. Oxygen anion voids are usually divided into GX, GGX, and γ types, among the GX type, X is usually Ser or threonine (Thr). In most cases, it contains a third amino acid, aspartic acid (Asp) or asparagine, which assist in stabilizing the oxygen anion pore through hydrogen bonding. GX type usually identifies lipases with medium-length carbon chains, while GGX typing identifies short-chain length-specific lipases and carboxylesterases^8,9^.

Lipase can catalyze a variety of reaction types, and the types of catalytic substrates are also varied, but different lipase-catalyzed reactions and substrates are greatly preferred^9–11^. Location-specific lipases refer to lipases that specifically recognize the ability of alpha-ester and beta-ester bonds on the two external positions of the glycerol ester skeleton^3,12^. Among them, the alpha-specific lipase mainly hydrolyzes the first and third triglyceride positions, which is called sn-1 and third position-specific lipase^13,14^. The composition of breast milk fat is very complicated, among which the fatty acid distribution of triglyceride has obvious specificity, approximately 70% of saturated fatty acids (mostly palmitic acid) are distributed in the sn-2 position, forming the main triglyceride type of ‘oleic acid-palm acid-oleic acid’^15,16^. At present, the fat of infant formula milk powder is mostly derived from vegetable oil. However, vegetable-derived saturated fatty acids are mostly distributed in the sn1,3 position of the glycerol skeleton. Therefore, the distribution of fatty acids in formula milk powder is quite different from that in breast milk^17^. Sn1,3-specific lipase is used to transesterify vegetable fat and reconstitute the position of intramolecular fatty acids to synthesize and reconstitute them^18^. Breast milk fat substitutes and formula milk powder, which are close to breast milk fat, have become a research hotspot in recent years^19^.

Castor (*Ricinus communis* L.) is a special oil crop, which belongs to the family Euphorbiaceae. Castor bean has active fatty acid metabolism and high specific lipase content. Research regarding lipase in castor has been conducted for decades^11,12,20,21^ cloned a lipase from castor oil. Here, a novel sn1,3-specific lipase was cloned from seeds of castor germinated for 4 days and expressed in *Pichia pastoris*. The purified lipase was characterized and the hydrolysis ability was studied to enrich the source of sn1,3 lipase in food and to improve the application value of castor in food.

## Materials and methods

### Strains, vectors, and media

Castor seeds Tongbi 5 (The certification number is Mengrenbi 2003003) were provided by the Castor Engineering Center of Tongliao City, Inner Mongolia, China. We ensure that we have permission to collect *Ricinus communis* L., and we confirm that the collection of *Ricinus communis* L. comply with relevant institutional, national, and international guidelines and legislation. *Escherichia coli* DH5α and PMD19-T carrier (for recombinant plasmid amplification) were purchased from TAKARA (TAKARA Biomedical Technology, Beijing, Co., Ltd.). *Pichia pastoris* X-33 and pPICZα-A were obtained from INVITROGEN as expression hosts and vectors, respectively.

LB medium (containing 0.5% yeast extract, w/v; 1% peptone, w/v; 1% NaCl, w/v) was used to culture *E. coli* with 100 μg/ml ampicillin or 25 μg/ml Zeocin. YPD-Zeocin plate (1% yeast extract, w/v; 2% peptone, w/v; 2% glucose, w/v; 2% agar, w/v; 100 μg/ml Zeocin) and buffered glycerol-complex medium (BMGY) (containing 1% glycerol, v/v; 2% peptone, w/v; 1% yeast extract, w/v; 1.34% YNB, w/v; 4 × 10–5% biotin, w/v; 100 mM potassium phosphate buffer, pH 6.0) were used for the growth and culture of *Pichia pastoris* yeast. Buffered methanol-complex medium (BMMY) (containing 0.5% methanol, v/v; 2% peptone, w/v; 1% yeast extract, w/v; 1.34% YNB, w/v; 4 × 10–5% biotin, w/v; 100 mM potassium phosphate buffer, pH 6.0) was used to induce lipase. Skerman’s basal mineral salt media (BSM) (containing 5.55% glycerol, w/v; 0.46% potassium hydroxide, w/v; 2.02% potassium sulfate, w/v; 0.10% calcium sulfate, w/v; 1.66% magnesium sulfate·7H_2_O, w/v; 2.71% tripotassium phosphate, v/v; 0.44% PTM1, v/v) was used for high-density fermentation. The fed-batch fermentation of the transformant was previously described^22^. Glycerol buffer or methanol induction medium (bmgy/bmmy) referred to Pichia Expression Kit (INVITROGEN Inc.).

### Reagents

LA-Taq DNA polymerase, restriction endonuclease, and T4 DNA ligase were purchased from TAKARA (TAKARA Biomedical Technology, Beijing, Co., Ltd.). P-nitrophenol palmitate (pnpp), p-NPC2, p-NPC5, p-NPC8, p-NPC12, p-NPC14, and all triglycerides were purchased from SIGMA (American). Castor oil was supplied by Inner Mongolia Tongliao XINGHE Biotechnology Co., Ltd. All chromatographic reagents (such as n-hexane), used for high performance liquid chromatography (HPLC), and analytical reagents were purchased from DINGGUO Biotechnology Co., Ltd. (Beijing, China).

### Cloning and sequence analysis of lipase gene

Total RNA was extracted from the castor bean using the MiniBEST Plant RNA Extraction Kit (9769S, TAKARA, China) following the manufacturer’s instructions. The extracted RNA was used as a template for the generation of First-strand cDNA using PrimeScript IV 1st Strand cDNA Synthesis Mix (6215A, TAKARA, China). The primers RLF1 (5′-CCATGGATGCTTAAGGCTTCCTAC-3′) and RLR1 (5′-CT CGAGATTGTTGTGCTCGAG-3′) used for the amplification of full-length lipase gene were designed according to castor genome data. The conditions for PCR were as follows: pre-denaturation for 5 min at 94 ℃ for 30 s, 55 ℃ for 30 s, 72 ℃ for 1 min, 30 cycles, extension for 5 min at 72 ℃. The Gel Recovery Kit (OMEGA) was used for electrophoresis detection, purification, and sequencing of *E. coli* DH5α with the pMD19-T vector.

Nucleotide and amino acid sequence alignment were performed using the Blast server (http://www.ncbi.nlm.nih.gov/blast). Molecular weight isoelectric point (pI) was estimated using the protparam tool (http://web.expasy.org/protparam/). Signal peptide was predicted on the signalP 4.1 server (http://www.cbs.dtu.dku/service/signalp/). Clustal Omega (http://www.ebi.ac.uk/tools/clustalw2/index.html) was used for multiple alignment analysis of amino acid sequences.

### Expression of lipase gene in *P. pastoris*

The pMD19-T-*RcLipase* and pPICZ a-A vectors were digested by *Nco*I and *Xho*I, respectively, and recombined using T4 DNA ligase. The recombinant plasmid was linearized by *Sac*I and transformed into *P. pastoris* X-33 by electroporation. The transformants were cultured on YPD-Zeocin agar plates for 2–3 days. Positive transformants were cultured in BMGY medium at 200 rpm at 30 ℃ until OD_600_ reached 2–6. Cells were collected and suspended in BMMY medium for 5 days. During this period, induction was maintained at a final concentration of 0.5% (v/v) every 24 h. The supernatant was obtained from crude enzyme solution (supernatant) after centrifugation at 10,000 rpm for 10 min, thereafter, lipase activity was detected.

### Fed-batch fermentation

In order to expand the production scale of lipase, a 3 L fermentation was adopted according to the method described^13^. Yeast seed liquid (100 ml) was added to BSM medium (900 ml). The fermentation process consisted of three phases, including the glycerol culture stage, glycerol feeding stage, and methanol induction stage. Glycerol culture was conducted at 30 ℃, with pH 6.0 maintained using NH_4_OH. The initial glycerol was depleted for about 20 h and entered the glycerol feeding stage. In the glycerol feeding stage, 50% glycerol (glycerol:water = 1:1, v/v) was added to the fermentation tank until OD_600_ reached 110. After the exhaustion of glycerol in the medium, it entered the methanol induction phase, where the temperature was maintained at 28 ℃, and the concentration of methanol was maintained at 0.1 ± 0.02% (w/v). The fermented samples were centrifuged at 10,000 rpm for 10 min. The cell number, lipase activity, and protein concentration of the supernatant were measured. The concentration of protein was determined using the Bradford method, and bovine serum albumin (BSA) (Beijing Solarbio Science & Technology Co., Ltd.) was used as the standard.

### Enzyme activity measurement

The activity of enzymes was determined using a spectrophotometer. The mother liquor of pNP (p-nitrophenol) with 20 mM was diluted into different concentration gradients at 40 ℃ for 15 min, and the termination reaction of ethanol with 500 μl 95% was added. The absorbance value was determined at 410 nm, and the standard curve was drawn. 2 ml EP tube was mixed with 300 ml buffer solution (0.3% p-nitrophenol palmitate, pNPP) isopropanol, 20 mM Tris–HCl buffer solution (pH 8.0, 0.11% gum arabic), 25 ml crude enzyme solution, 20 mM Tris–HCl buffer solution (pH 8.0, 0.11% gum arabic), and 40 mM Tris–HCl buffer solution (25 ml, 0.11% gum arabic), respectively. The reaction was terminated by adding 500 μl 95% ethanol at 15 min. The absorbance value was determined at 410 nm, and the concentration of pNP was obtained according to the standard curve, followed by enzyme activity calculation. The amount of p-nitrophenol required to catalyze the production of p-nitrophenol by substrates within 1 min is defined as one unit of enzyme activity (U).

### Biochemical characterization of purified enzyme

#### Effect of pH on RcLipase activity and stability

To determine the optimum lipase pH, pNPP activity was measured under the optimum reaction temperature and concentration of 20 mM buffer with various pH values (pH 4.0–9.0) for 15 min. All experiments were repeated three times. The effect of pH on lipase activity was expressed by relative enzyme activity. The maximum enzyme activity was determined to be 100%.

For the lipase pH stability test, lipase protein samples were placed in 100 mM buffer with different pH and incubated at 40 ℃ for 12 h to determine the enzyme activity. The effect of pH on the stability of lipase was expressed by relative enzyme activity. The enzyme activity of untreated protein samples was determined to be 100%.

#### Effect of temperature on RcLipase activity and stability

Lipase activity was determined at 30, 35, 40, 45, 50, 55, 60, and 70 ℃ with pNPP as substrate in 20 mM Tris–HCl (pH 8.0) buffer solution for 15 min. All experiments were repeated three times. The effect of temperature on lipase activity was expressed by relative enzyme activity. The maximum enzyme activity was determined to be 100%.

For the determination of temperature stability of lipase, the lipase protein samples were incubated at 30, 40, 50, 60, and 70 ℃ for 12 h, and the enzyme activity was determined immediately after being cooled with ice water every 6 h. The effect of temperature on the stability of lipase was expressed by relative enzyme activity. The enzyme activity of untreated protein samples was determined to be 100%.

### Effect of metal ions, surfactants, and organic solvents on RcLipase activity

#### Effect of metal ions on RcLipase activity

NiCl_2_, ZnSO_4_, MnSO_4_, MgSO_4_, FeCl_2_, CaCl_2_, CoCl_2_, and CuCl_2_ were added to the lipase protein samples, and the final concentration of metal ions in the system was 5 mM. The mixtures were incubated at 40 ℃ for 2 h and the activity of lipase was determined with pNPP as the substrate under the optimum reaction conditions. All experiments were repeated three times. The effect of metal ions on lipase activity was expressed by relative enzyme activity. The activity of lipase was determined to be 100% in the absence of metal ions.

#### Effect of surfactants on RcLipase activity

SDS, Tween 20, Tween 60, Tween 80, and Triton X-100 were added to the lipase protein samples, respectively, and the final concentration of surfactant in the system was 1% (m/v). The mixtures were incubated at 40 ℃ for 2 h and the activity of lipase was determined with pNPP as the substrate under the optimum reaction conditions. All experiments were repeated three times. The effect of surfactant on lipase activity was expressed by relative enzyme activity. The activity of lipase determined by protein samples without surfactant was 100%.

#### Effect of organic solvents on RcLipase activity

Methanol, ethanol, isopropyl alcohol, acetone, toluene, and chloroform were added to the lipase protein samples, respectively, and the final concentration of organic solvent was 30% (v/v). The mixtures were incubated at 40 ℃ for 2 h and the enzyme activity was determined with pNPP as the substrate under the optimum reaction conditions. All experiments were conducted three times. The effect of organic solvents on lipase activity was expressed by relative enzyme activity. The activity of lipase was determined to be 100% in the samples without organic solvents.

### Substrate specificity of RcLipase

The purified lipase (100 mg) was reacted with different substrates using 20 mM Tris–HCl (pH 8.0) at 40 ℃ for 10 min to detect the specificity of lipase for substrates. The p-nitrophenol ester including p-NPC2, p-NPC4, p-NPC6, p-NPC8, p-NPC10, p-NPC12, p-NPC14, and p-NPC16 were used as substrates. The triglycerides, including triacetin (C2), tributyrin (C4), tricaproin (C6), tricaprylin (C8), tricaprin (C10), trilaurin (C12), trimyristin (C14), tripalmitin (C16), triolein (C18), and castor oil (C18) were also used as substrates. P-nitrophenol was determined using the nitrophenol method, and triglycerides were determined using HPLC.

### Regiospecific analysis

The determination of the regiospecificity of RcLipase was performed according to a previously described method^22^, In brief, triolein (500 mg) and 500 μl RcLipase (50 U) were dissolved in 20 ml of 20 mM Tris HCl (pH 8.0), the mixture was shaken at 180 rpm for 12 h at 40 ℃. Samples (0.05 ml) were withdrawn periodically and centrifuged at 10,000 rpm for 5 min, 0.02 ml upper layer was transferred into another centrifugation tube and was mixed with 0.5 g anhydrous sodium sulfate and 1 mL of n-hexane, 2-propanol and methanoic acid (20:1:0.003, by volume) were added and centrifuged at 10,000 rpm for 10 min. The supernatant was used for HPLC.

### Determination of kinetic parameters

The kinetic parameters of lipase were determined using glycerol ester substrates (triolein, tripalmitate, and castor oil). The substrate concentration range was controlled from 0 to 1 mM, and the reaction conditions were 40 ℃, 20 mM Tris–HCl buffer with pH 8.0. The kinetic parameters *V*_*max*_, *K*_*m*_, and *K*_*cat*_ of the lipase enzymatic reaction were calculated using the non-linear regression and Michaelis–Menten equation.

### RcLipase-catalyzed synthesis of ‘oleic-palmitic-oleic’

RcLipase dry powder was mixed with a mixture of palmitic acid triglyceride (100 mg) and oleic acid (molar ratio 1:2, 1:4, 1:6, 1:8), so that the final concentration of the enzyme is 2%, 4%, 6%, 8%, 10%, and 12%. N-hexane (3 ml) was used as a solvent to catalyze the mixture for 2, 4, 6, 8, 10, 12, and 24 h at 40 ℃. The content of oleic acid and palmitic acid was determined using HPLC as described^22^.

### Statistical analysis

All experiments were repeated in triplicate. Data analytical variance was presented as means ± standard errors using SPSS version 19.0 (SPSS Inc., Chicago, IL). One-way analysis of variance (ANOVA) was used to analyze the data. Origin 8.0 software was used for hyperbl non-linear regression analysis.

## Results

### Cloning and sequence analysis of a novel lipase gene from *Ricinus communis* L.

**Figure 1:**
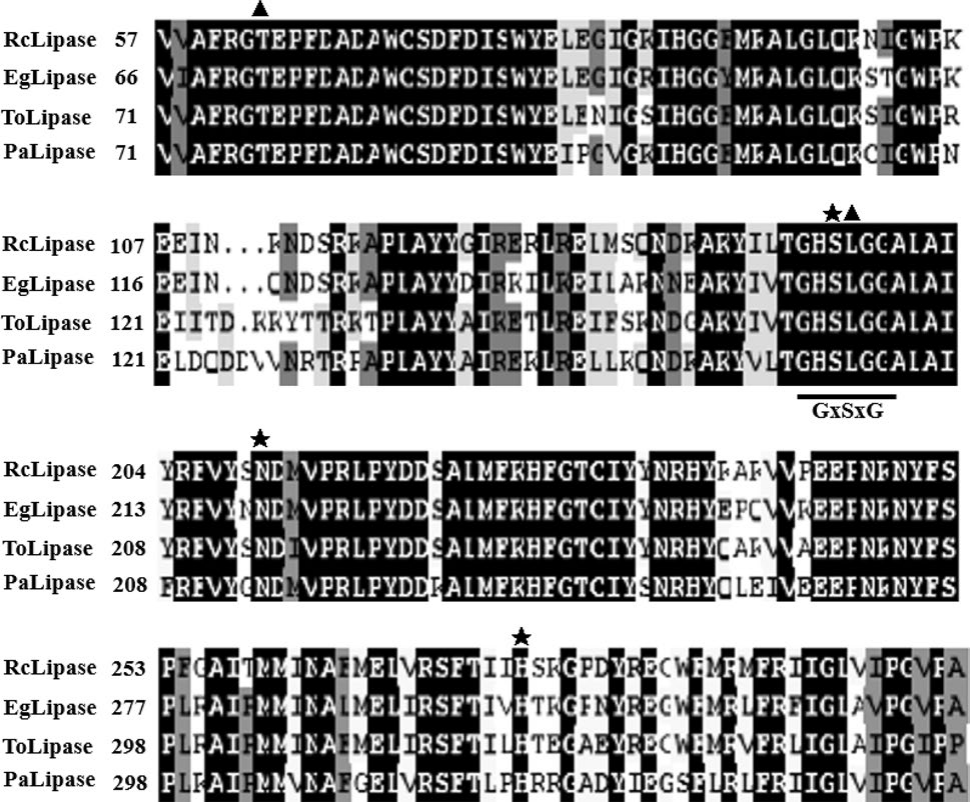
RcLipase is aligned with the amino acid sequence of other lipases. The number on the left is the first amino acid residue in each row. The abbreviations and login numbers of these lipases are as follows: *Eucalyptus grandis* (EgLipase, XP_018716435.1), *Trema orientale* (ToLipase, POO02811.1), *Parasponia andersonii* (PaLipase, PON39785.1). The same residues are colored black and the conservative amino acid residues are colored grey. Conservative ester box GxSxG is underlined. Three residues Ser (146 site), Asp (211 site), and His (276 site) from the catalytic triad are identified by the filled pentagram. Threonine (63 site) and leucine (147 site) are identified by filled triangles.

The ORF of the *RcLipase* gene was 987 bp, without introns. It encodes 329 amino acids and does not contain signal peptides (Fig. S1). The molecular weight of the predicted mature protein was 38.24 KDa and the inferred pI was 8.47. The multiple comparison between *RcLipase* and other lipases showed that the highly conserved catalytic trimer consisted of Ser (146 site), Asp (211 site), and His (276 site). RcLipase had a conservative ester box GxSxG, which conformed to the conservative domain structure of lipase in class III (Fig. 1). There were Gly-X1-Ser-X2 oxygen ion holes, X1 was threonine (63 site), X2 was leucine (147 site).

### Expression and purification of RcLipase

**Figure 2:**
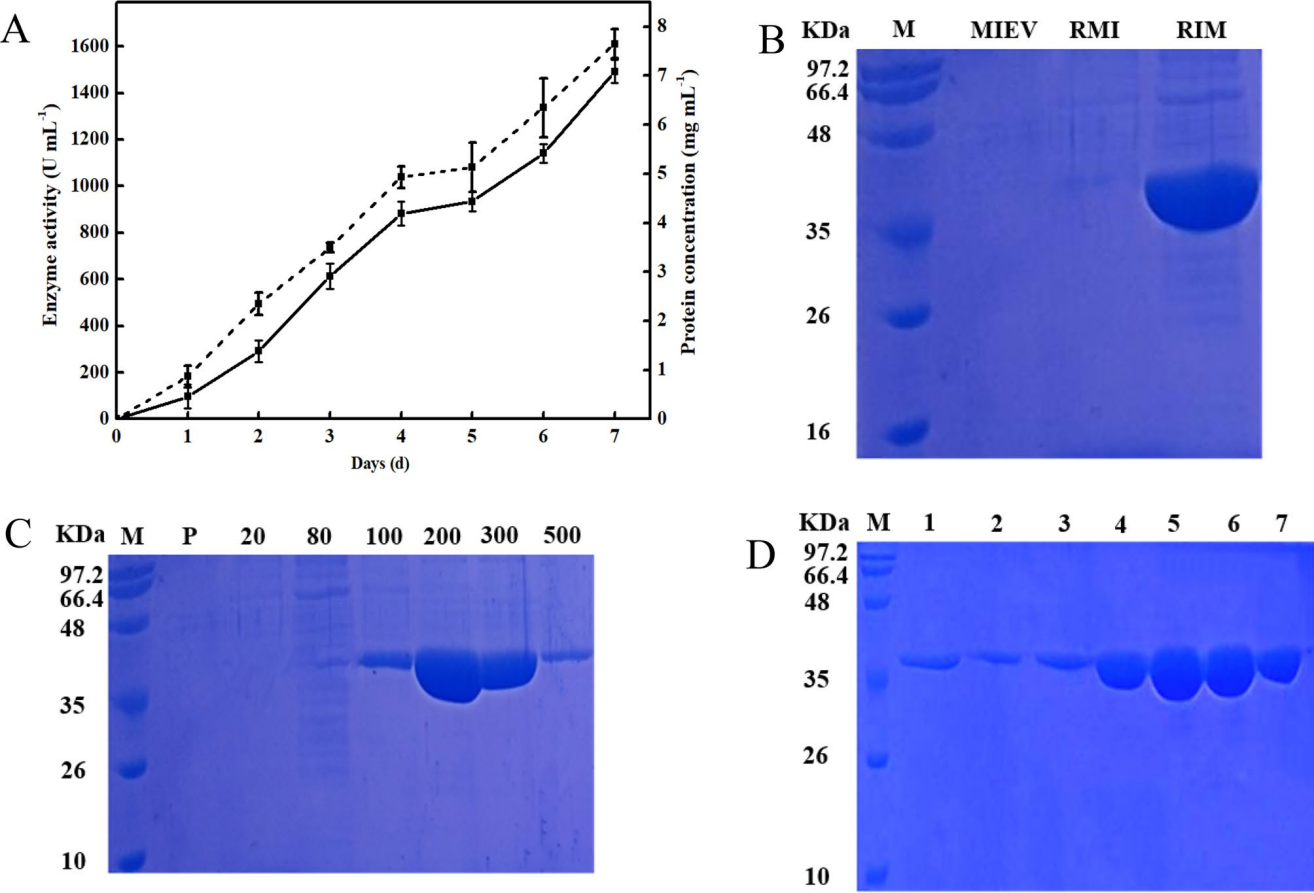
Expression and purification of RcLipase. (**A**) Expression and purification of RcLipase in *Pichia pastoris* in a 3 l fermentor. Solid line, enzyme activity; dotted line, protein concentration. (**B**) RcLipase in *Pichia pastoris* was detected using SDS-PAGE. M, protein molecular weight marker (band size is indicated on the left); *MIEV* methanol-induced empty vector supernatant, *RMI* 7 days supernatant of RcLipase without methanol induction; RIM, 7 days supernatant of RcLipase induced by methanol. (**C**) The result of RcLipase affinity chromatography purified using SDS-PAGE. M, protein molecular weight marker (band size is indicated on the left); P, through out; 20, 20 mM imidazole elution; 80, 80 mM imidazole elution; 100, 100 mM imidazole elution; 200, 200 mM imidazole elution; 300, 300 mM imidazole elution; 500, 500 mM imidazole elution. (**D**) RcLipase molecular sieve chromatography purified SDS-PAGE results. M, protein molecular weight marker (band size is indicated on the left); 1–7, tubules isolated from protein samples.

RcLipase was expressed in *Pichia pastoris*. After 7 days of fermentation in a 3 l fermentor, the extracellular lipase activity reached 1492 U/ml (for pNPP) and the protein concentration was 7.65 mg/ml (Fig. 2A). After purification using affinity chromatography, the recovery of crude enzyme was 80.2%, and the specific activity was 147.2 U/mg. The purified RcLipase was detected using SDS-PAGE with a band at 38 KDa (Fig. 2B–D).

### Biochemical characterization of the purified enzymes

**Figure 3:**
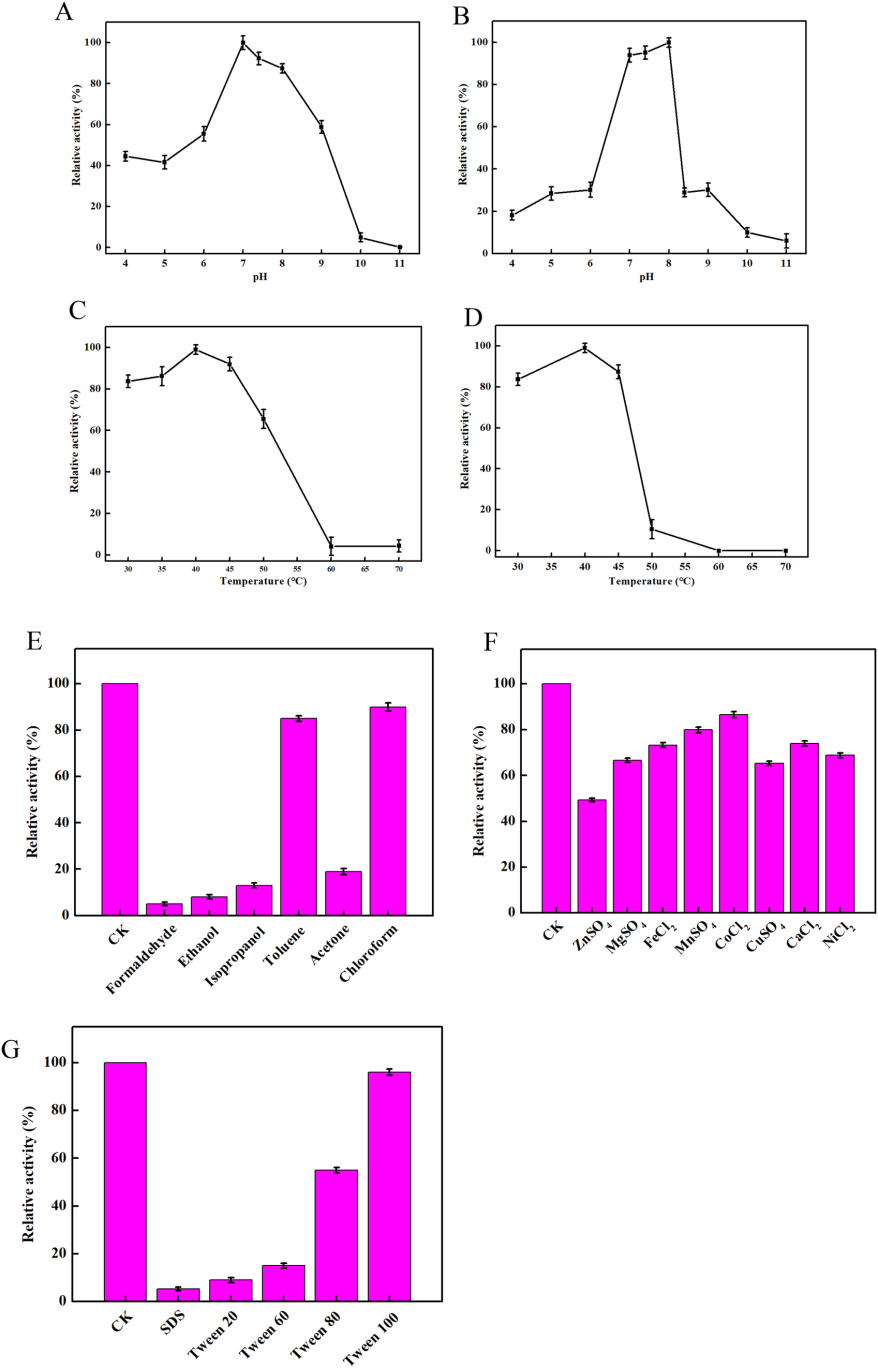
The biochemical characterization of purified RcLipase enzyme. (**A**) The optimal pH. (**B**) pH stability. (**C**) Optimal temperature. (**D**) Thermostability of RcLipase. (**E**) Optimal organic solvents. (**F**) Optimal Metal ions. (**G**) Effects of organic solvents on lipase activity.

As shown in Fig. 3A, the optimum pH of RcLipase was 8.0. To determine pH stability, the enzyme was incubated in various buffers at 40 °C for 12 h, and the residual activity was measured. Buffers used: citrate, pH 3.0–6.0; phosphate, pH 6.0–8.0; Tris–HCl, pH 8.0–10.0; CAPSO (cyclohexylamino)-1-propanesulphonic acid), pH 10.0–11.0. The activity of RcLipase remained more than 90% after 24 h of treatment at pH 7.0–8.0, and 60% after 12 h of treatment at pH 8.0–9.0, indicating that the enzyme was tolerant to alkali (Fig. 3B).

To determine the optimal temperature of RcLipase, the enzyme activity was assayed at temperatures ranging from 30 to 70 ℃ in 20 mM Tris–HCl buffer (pH 8.0) for 15 min. The relative activity of RcLipase was the strongest at 40 ℃ (Fig. 3C). To determine the thermostability of RcLipase, the enzyme activity was assayed at temperatures ranging from 30 to 70 ℃ in 20 mM Tris–HCl buffer (pH 8.0) for 12 h. After incubation at 40–45 ℃ for 12 h, it retains more than 80% of its relative activity; after incubation at 50 ℃ for 12 h, the relative activity drops to 13.57%; after incubation at 60–70 ℃ for 12 h, the activity is completely lost. Therefore, the enzyme is stable below 40–45 ℃ (Fig. 3D).

Formaldehyde and ethanol exerted significant inhibitory effects on RcLipase. The activity of RcLipase decreased by 88.2% and 88.4% in 2 h, followed by isopropanol and acetone. The activity of RcLipase decreased by 77.2% and 78.7% in 2 h, respectively. Chloroform and toluene exerted the lowest effect (10% and 15%, respectively) (Fig. 3E).

The inhibitory effects of 0.1% SDS, Tween20, Tween60, and Tween80 on RcLipase were obvious. After 2 h, the activity of RcLipase decreased by 95.1%, 91.8%, 85.3%, and 45%, respectively. Triton X-100 had no obvious inhibitory effect on RcLipase (Fig. 3F).

The inhibitory effect of Zn^2+^ and Cu^2+^ ions on RcLipase was obvious. After 2 h, the activity of RcLipase decreased by 53% and 40%, respectively. However, the inhibitory effect of other metal ions on RcLipase was not obvious (Fig. 3G).

### Substrate and position specificity of RcLipase

As shown in Table 1, the enzyme displays hydrolytic activity to both pNPC and natural lipids, but both display hydrolytic activity to pNPC and natural lipids. The hydrolysis activity of long-chain substrates (C14/C16/C18) was stronger than that of short-chain substrates. The hydrolysis activity of two basic lipids, tripalmitic glycerides, and trioleic glycerides, was also strong for artificial breast milk (88.35% and 90.1%). This indicated that the enzyme had the potential to synthesize artificial breast milk lipids.

**Table 1:** Substrate specificity of RcLipase.

Substrate	Specific activity (U/ml)	Relative activity (%)
pNPA (p-nitrophenyl acetate) (C_2_)	42.062 ± 1.45	10.01
pNPB (p-nitrophenyl butyrate) (C4)	47.902 ± 2.30	11.4
pNPH (p-nitrophenyl hexanoate) (C6)	140.473 ± 2.13	33.43
pNPC (p-nitrophenyl caprylate) (C8)	211.445 ± 4.34	50.32
pNPD (p-nitrophenyl decanoate) (C10)	280.983 ± 3.10	66.71
pNPL (p-nitrophenyl laurate) (C12)	317.711 ± 3.28	75.43
pNPM (p-nitrophenyl myristate) (C14)	328.536 ± 2.88	78.32
pNPP (p-nitrophenyl palmitate) (C16)	421.201 ± 5.32	100
Triacetin (C2)	44.223 ± 1.13	8.7
Tributyrin (C4)	68.368 ± 2.34	13.45
Tricaproin (C6)	185.177 ± 0.78	36.43
Tricaprylin (C8)	281.238 ± 3.11	55.328
Tricaprin (C10)	378.345 ± 4.37	74.432
Trilaurin (C12)	383.418 ± 2.11	75.43
Trimyristin (C14)	412.290 ± 4.55	81.11
Tripalmitin (C16)	498.706 ± 5.37	86.11
Triolein (C18)	508.310 ± 3.83	90.1
Castor oil (C18)	564.73 ± 2.73	100

In order to study the location specificity, the hydrolysate was analyzed using high performance liquid chromatography with RcLipase as a catalyst (Table 1). The results showed that it could hydrolyze triglycerides to produce 1, 2(2, 3)-DAG and 1,3-DAG. The ratio of 1, 2(2, 3)-DAG to 1,3-DAG in the hydrolysate was 24.52, 16.54 and 8.52 in 10 min, 1 h, and 4 h, respectively, for sn-1,3 regiospecific lipases, the reported ratios of 1, 2(2, 3)-DAG to 1,3-DAG hydrolysis generally range from 7 to 26.71^22^. The agreement with previous studies indicates that that RcLipase has sn1,3-specific lipase.

### Determination of kinetic parameters

Due to the need to synthesize oleic acid-palmitic acid-oleic acid, the enzymatic kinetic parameters of trioleic acid glyceride and tripalmitic acid glyceride were analyzed by RcLipase. The lower the km, the stronger the affinity between the enzyme and the substrate. The higher the catalytic activity, the stronger the catalytic activity of *K*_cat_/*K*_m_. As shown in Table 2, RcLipase exhibited *K*_m_ and *K*_cat_ values towards tripalmitin of 0.17 ± 0.01 mM and 36.81 ± 5.94 s^−1^, respectively; RcLipase exhibited *K*_m_ and *K*_cat_ values towards triolein of 0.21 ± 0.01 mM and 36.46 ± 4.89 s^−1^, respectively. The two parameters in the substrate of castor oil displayed no significant difference between tripalmitin and triolein, although they displayed a 1.34- and 1.68-fold decrease in the *K*_cat_/*K*_m_ values compared to the castor oil substrates. The non-linear regression curve analysis of RcLipase with different substrates were shown in Fig. S2. Results showed that RcLipase displayed high affinity and catalytic activity to triolein and tripalmitate, which further indicates that RcLipase can be used as an enzyme to produce artificial breast milk lipids.

**Table 2:** The kinetic parameters of RcLipase.

Protein	Substrate	*K*_m_	*V*_max_ (mmol/mg_enzyme_/min)	*K*_*cat*_ (s^−1^)	*K*_cat_/*K*_m_
RcLipase	Tripalmitin	0.17 ± 0.01(mM)	1885.27 ± 50.21	36.81 ± 5.94	216.52 ± 12.51 (s^-1^ mM^-1^)
	Triolein	0.21 ± 0.01 (mM)	1875.16 ± 44.67	36.46 ± 4.89	172.62 ± 9.72 (s^-1^ mM^-1^)
	Castor oil	0.13 ± 0.01 (mM)	1903.23 ± 39.36	37.63 ± 6.33	289.46 ± 8.37 (s^-1^ mM^-1^)

Based on the non-linear regression, the Michaelis constant (*K*_m_), maximum reaction velocity (*V*_max_), and catalytic rate constant (*k*_cat_) for the substrates tripalmitin, triolein, castor oil were determined, respectively. Hydrolytic reactions occurred at a pH of 8.0.

### Synthesis of “oleic-palmitic-oleic”

#### Effect of substrate ratio on incorporation of oleic acid and stability of 2-palmitic acid

**Figure 4:**
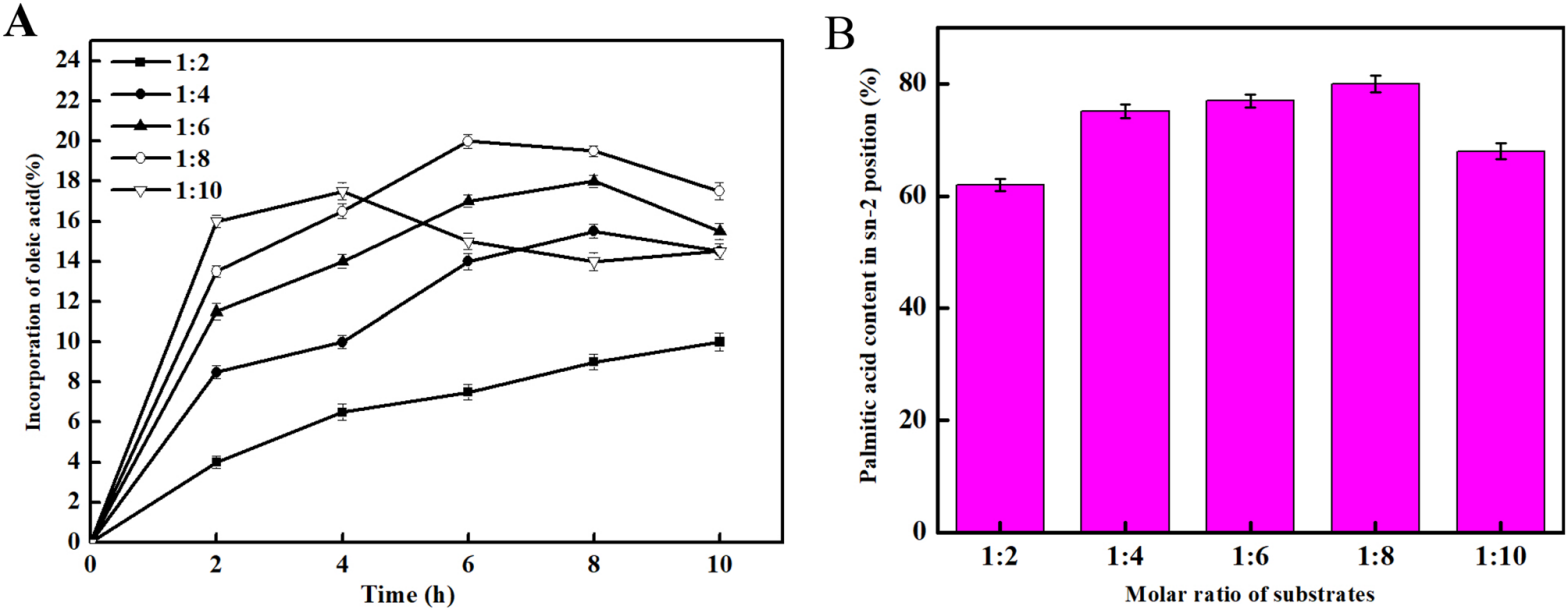
Effect of substrate ratio on incorporation of oleic acid and stability of 2-palmitic acid. (**A**) Oleic acid incorporation with various substrate ratios. (**B**) Stability of 2-palmitic acid with varying substrate ratios.

We attempted synthesis of oleic acid-palmitic acid-oleic acid and compared the conditions of the reaction process. It was found that the highest incorporation of oleic acid was 19.5% at the substrate concentration ratio (oleic acid: triglyceride) of 1:8 for 6 h, and the lowest migration of palmitic acid at sn-2 position was 77.6% at the concentration of 1:8 for 6 h (Fig. 4A,B).

#### Effect of reaction time on incorporation of oleic acid and stability of 2-palmitic acid

**Figure 5:**
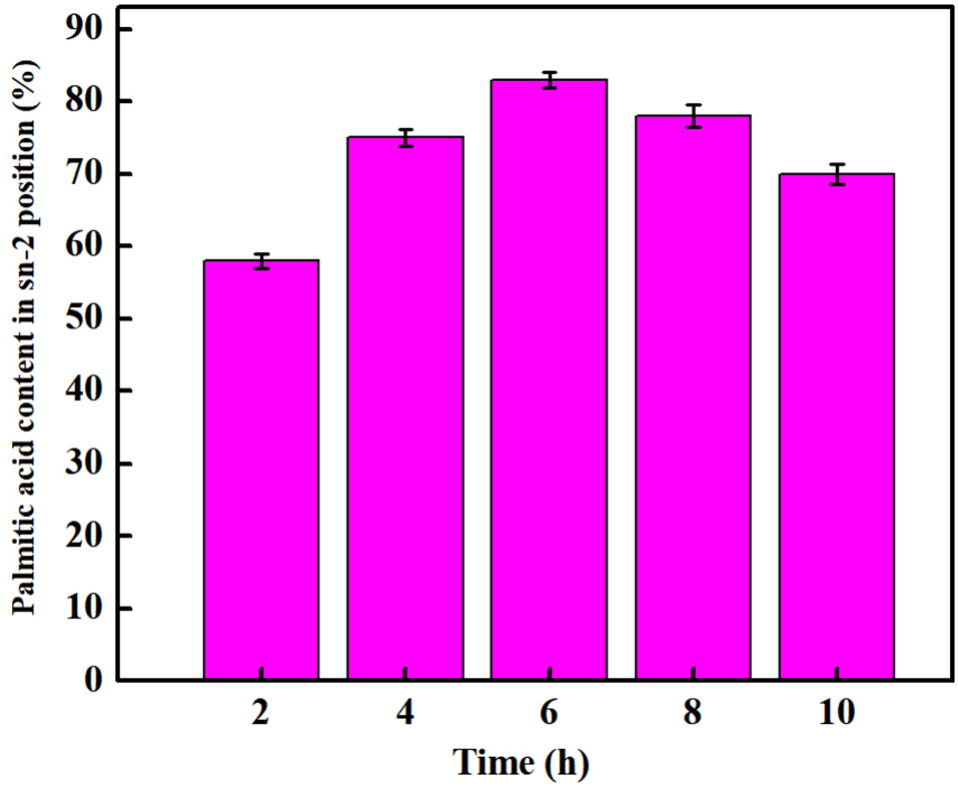
The stability of sn-2 palmitic acid at different time periods.

Based on our results, we measured the stability of sn-2 palmitic acid at various time periods. As shown in Fig. 5, when the substrate ratio is 1:8, the stability of sn-2 palmitic acid was 78.1% in 6 h. The result showed that oleic acid was the most stable at 6 h.

#### Effect of enzyme levels on incorporation of oleic acid and stability of 2-palmitic acid

**Figure 6:**
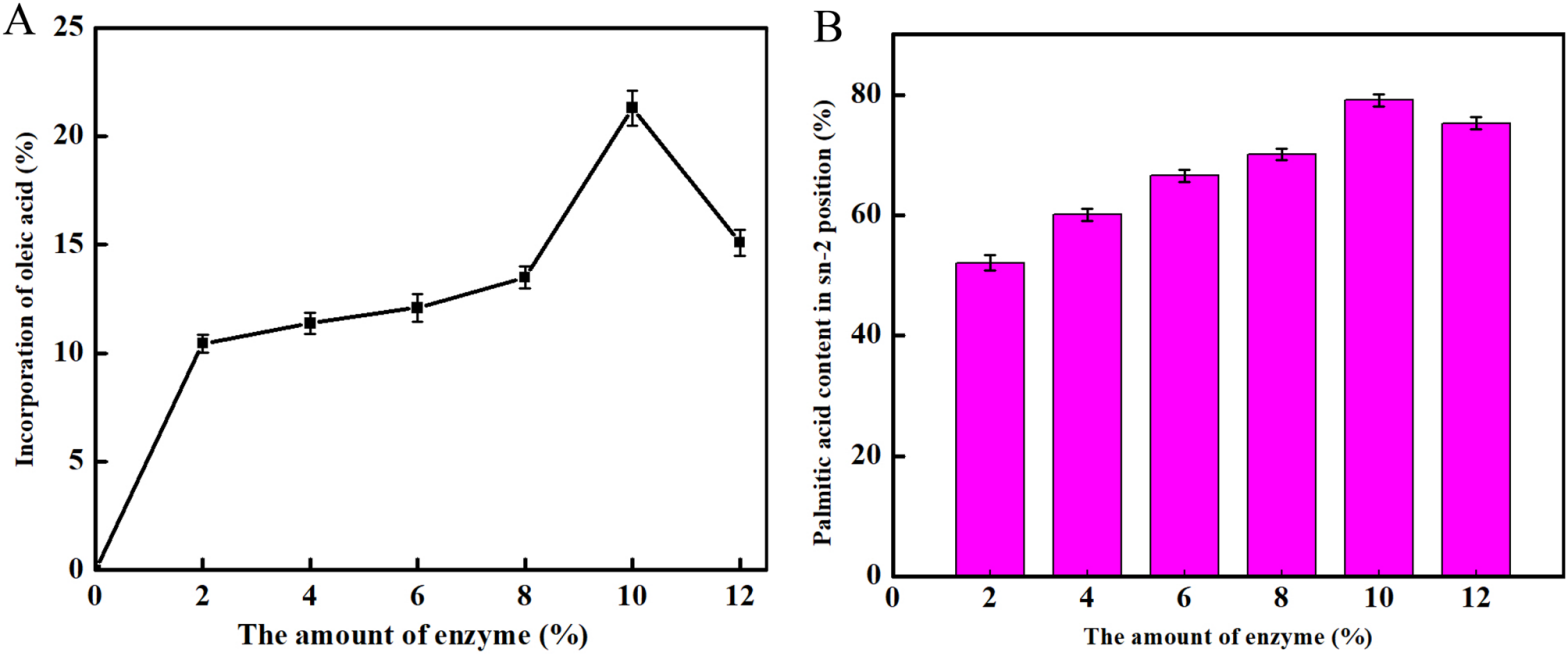
Effect of enzyme levels on incorporation of oleic acid and stability of 2-palmitic acid. (**A**) Oleic acid incorporation with varying enzyme levels. (**B**) Stability of 2-palmitic acid with varying enzyme levels.

As shown in Fig. 6, when the enzyme concentration was 10%, the incorporation rate of oleic acid was the highest (21.3%) and the palmitic acid was the most stable (79.1%). Therefore, the optimal enzyme concentration was 10%.

## Discussion

It is reported that many germinating seeds contain large amounts of lipase, which is mainly considered to hydrolyze triglycerides (TAG) and release carbon skeletons for seed germination and growth^23^. A new lipase gene (*RcLipase*) was cloned from Castor seeds and expressed as an extracellular protein in *Pichia pastoris*. After the homology comparison, it was found that there were Gly-X1-Ser-X2 oxygen ion holes and a conservative ester box GxSxG, which agreed with the conservative region structure of lipases in class III, which are widely distributed in plants, animals, and prokaryotes. They can hydrolyze long chain acyl triglycerides to binary glycerides and free fatty acids at the water/lipid interface^24^. RcLipase is a lipase in the class III lipase superfamily identified from castor.

RcLipase in *Ricinus communis* was cloned from seeds, which were germinated for 2 d. The optimum pH value was 8.0, which was lower than that of *Thermomyces lanuginosus* TlLipase (pH 9.0)^25^ and higher than that of *Malbranchea cinnamea* McLipase (pH 7.5)^26^ and *Neosartorya fischeri* NfLipase (pH 5.0)^27^. Moreover, the optimum temperature of RcLipase was 40 ℃, which was lower than that of McLipase (45 ℃)^26^ and TtLipase (60 ℃) from *Talaromyces thermophilus*^28^.

Most lipases showed high catalytic activity towards medium and long chain substrates, such as McLipase (pNPM, C14)^26^ and *N. fischeri* lipase (pNPL, C12)^27^. RcLipase showed higher hydrolytic activity to natural fatty acids, such as castor oil (C9), palmitic acid (C16), and oleic acid (C18). It also showed strong hydrolytic activity for pNPC8, pNPL12, and pNPM14, indicating that the enzyme was a medium long chain neutral lipase. *K*_*m*_ is an important kinetic parameter of the enzyme, which indicates the ratio of the disappearance rate constant to the formation rate constant of the ES complex. The reciprocal of *K*_*m*_ (1/*K*_*m*_) refers to the affinity of substrate to enzyme. RcLipase is 5.88 mm/min for 1/*K*_*m*_ of palmitate, which is higher than *Pseudomonas* lipase (0.7 mM). The overall efficiency of the *K*_cat_/*K*_m_ catalyst was 275.36 (s^−1^ mM^−1^) for the reaction of palmitate, which is higher than *Staphylococcus xylosus* SxLipase (164.4 s^-1^ mM^−1^)^29^. This shows that the enzyme exerts good hydrolytic activity for palmitate and oleate, which are important substrates for the synthesis of artificial human milk fat.

The main type of triglyceride, “oleic-palmitic-oleic”, is formed by the fatty components of human milk, In recent years, it has become a research hotspot to select sn1,3-specific lipase for transesterification of vegetable oil and recombination of intramolecular fatty acid position to synthesize breast milk fat substitutes and formulas similar to breast milk fat^19^. At present, many lipases with sn1,3 site specificity have been cloned from microorganisms, such as *Rhizomucor miehei* RmLipase^30^ and *Malbranchea cinnamea* McLipA, which are sn1,3-specific lipases^26^. Most lipases from *G. candidum* are sn1,3-specific^31^. In general, the ratio of 1, 2(2, 3)-DAG to 1,3-DAG in the hydrolysate of triglyceride is used to evaluate the specificity of lipase location. For the lipase with sn1,3 site specificity, the ratio is usually 3.28–31.83^13,28^. The ratio of 1,2-DAG to 1,3-DAG is 10.52–26.81, which indicates that RcLipase displays sn1,3 regiospecificity, with glycerides and oleic acid as substrates, and 10% of the substrate concentration of RcLipase, the molar ratio of the substrate is 1:8 and the reaction time is 6 h. The composition of fatty acids in the reaction product is 19.5% oleic acid and 79.1% palmitic acid at sn-2. Therefore, RcLipase can potentially be used to synthesize human milk fat (oleic acid palmitate oleic acid). These results provide a reference for enriching sources of sn1,3 lipase and improving the application value of castor. Regional specificity of lipase depends on many factors, such as conserved pentapeptide residues and catalytic site distance between serine, residues of active sites^13^, size and/or ratio of affinity, and sparsity of binding sites^32^. Detailed mechanisms regarding RcLipase region specificity requires further study. In addition, both hydrolyzate products alcohols and carboxylic acids can act as enzyme inhibitors, and the in-depth study of this part will have a strong significance for the subsequent industrial production of the enzyme. Furthermore, our future research goal is to enhance the regional specificity of RcLipase to prepare products with high infant nutritional value. Therefore, in the future, it is necessary to study the reverse esterification reaction in order to have irreversible proof of this mechanism.

## Conclusions

This study provides the catalytic properties of lipase from castor in hydrolysis reactions for the production of oleic acid-palmitic acid-glycerol oleate. A neutral lipase was successfully expressed in *Pichia pastoris* X-33. The lipase belongs to the lipase III superfamily and displays sn1,3 regiospecificity. Furthermore, RcLipase exhibited high affinity and hydrolytic activity to the substrates of tripalmitin and triolein. Overall, RcLipase has the potential to be used to synthesize human milk fat (oleic palmitic acid oleic acid).

## Supplementary Information

Supplementary Information.

## Abbreviations

Symbol: Abbreviation meaning
RcLipase: Sn1,3-specific lipase from Castor bean
SDH: Conserved catalytic center ‘Serine-Aspartic acid-Histidine
GXSXG: Conserved pentapeptide ‘glycine-amino acid residues–Serine–amino acid residues–glycine’
GX: Conserved pentapeptide ‘glycine–amino acid residues’
GGX: Conserved pentapeptide ‘glycine–glycine–amino acid residues’
HPLC: High performance liquid chromatography
ORF: Open reading frame
PNPP: P-nitrophenyl palmitate
SDS-PAGE: Sodium dodecyl sulfate polyacrylamide gel electrophoresis
CAPSO: Cyclohexylamino-1-propanesulphonic acid
pNPC: P-nitrophenyl caprylate
pNPM: P-nitrophenyl myristate
pNPL: P-nitrophenyl laurate
ES: Enzyme and substrate
1,2-DAG: 1,2-Diacylglycerol
1,3-DAG: 1,3-Diacylglycerol
YNB: Yeast nitrogen base
LB: Lysogeny broth
BMGY: Buffered glycerol-complex medium
BMMY: Buffered methanol-complex medium
BSM: Buffered methanol-complex medium
PTM1: Microelement
pNP: P-nitrophenol
p-NPC2: P-nitrophenyl acetate
p-NPC5: P-nitrophenyl butyrate
p-NPC8: P-nitrophenyl caprylate
p-NPC10: P-nitrophenyl decanoate
p-NPC12: P-nitrophenyl laurate
p-NPC14: P-nitrophenyl myristate
YPD: Yeast extract peptone dextrose medium
BSM: Base medium
EP: Eppendorf
TLS detector: Thermoluminescent sheet stack

## References

[1] Naveen P, Rai DS, Shahane S, Mishra U (2018). Lipases: Sources, production, purification, and applications. Recent Pat. Biotechnol..

[2] Sarmah N, Revathi D, Sheelu G, Rani KY, Sridhar S, Mehtab V, Sumana C (2018). Recent advances on sources and industrial applications of lipases. Biotechnol. Prog..

[3] Casas-Godoy L, Gasteazoro F, Duquesne S, Bordes F, Marty A, Sandoval G (2018). Lipases. Methods Mol. Biol..

[4] Gupta R, Kumari A, Syal P, Singh Y (2015). Molecular and functional diversity of yeast and fungal lipases: Their role in biotechnology and cellular physiology. Prog. Lipid Res..

[5] Castillo GDV, Azcurra AI, Sotomayor CE (2019). Candida lipases: A review onbiochemical, molecular and pathogenic aspects. Rev. Fac. Cien Med. Univ. Nac. Cordoba.

[6] Hasan FSA, Hameed A (2006). Industrial applications of microbial lipases. Enzyme Microb. Technol..

[7] de Araújo ME, Franco YE, Messias MC, Longato GB, Pamphile JA, Carvalho PD (2017). Biocatalytic synthesis of flavonoid esters by lipases and their biological benefits. Planta Med..

[8] Fischer M, Thai QK, Grieb M, Pleiss J (2006). DWARF-a data warehouse system for analyzing protein families. BMC Bioinform..

[9] Kunduru KR, Basu A, Haim Zada M, Domb AJ (2015). Castor oil based biodegradable polyesters. Biomacromol.

[10] Maeshima M, Beevers H (1985). Purification and properties of glyoxysomal lipase from castor bean. Plant Physiol..

[11] Ory RL, Yatsu LY, Kircher HW (1968). Association of lipase activity with the spherosomes of *Ricinus communis*. Arch. Biochem. Biophys..

[12] Peter JE (2004). Cloning and characterization of the acid lipase from castor beans. Biol. Chem..

[13] Zhao Z, Hou S, Lan D, Wang X, Wang Y (2017). Crystal structure of a lipase from *Streptomyces* sp. strain W007: Implications for thermostability and regiospecificity. FEBS J..

[14] El-Alaoui M, Soulère L, Noiriel A, Queneau Y, Abousalham A (2017). α-eleostearic acid-containing triglycerides for a continuous assay to determine lipase, sn-1 and sn-3 regio-preference. Chem. Phys. Lipids.

[15] Jung HP, Kyung-Min P (2018). Cloning and protein expression of the sn-1(3) Regioselective lipase from *Cordyceps militaris*. Enzyme Microb. Technol..

[16] Yamamoto Y, Yoshida H, Nagai T, Hara S (2018). Preparation of chiral triacylglycerols, sn-POO and sn-OOP, via lipase-mediated acidolysis reaction. J. Oleo Sci..

[17] Jennings BH, Akoh CC (2000). Lipase-catalyzed modification of rice bran oil to incorporate capric acid. J. Agric. Food Chem..

[18] Ferreira-Dias S, Natália MO, Carla T (2018). Lipase-catalyzed synthesis of structured lipids at laboratory scale. Methods Mol. Biol..

[19] Wang J, Liu X (2016). Selective synthesis of human milk fat-style structured triglycerides from microalgal oil in a microfluidic reactor packed with immobilized lipase. Bioresour. Technol..

[20] Ory RL, Angelo AJS, Altschul AM (1960). Castor bean lipase. Action on its endogenous substrate. J. Lipid Res..

[21] Ory RL, Barker RH, Boudreaux GJ (1964). Nature of the cofactor for the acid lipase of *Ricinus communis*. Biochemistry.

[22] Duan XJ, Xiang M, Wang L, Yan QJ, Yang SQ, Jiang ZQ (2019). Biochemical characterization of a novel lipase from *Malbranchea cinnamomea* suitable for production of lipolyzed milkfat flavor and biodegradation of phthalate esters. Food Chem..

[23] Patui S, Clincon L, Peresson C (2014). Lipase activity and antioxidant capacity in coffee (*Coffea arabica* L.) seeds during germination. Plant Sci..

[24] Cerk IK, Wechselberger L, Oberer M (2018). Adipose triglyceride lipase regulation: An overview. Curr. Protein Pept. Sci..

[25] Zheng YY, Guo XH, Song NN, Li DC (2011). Thermophilic lipase from *Thermomyces lanuginosus*: Gene cloning, expression and characterization. J. Mol. Catal. B-Enzyme.

[26] Tong X, Busk PK, Lange L (2016). Characterization of a new sn-1, 3-regioselective triacylglycerol lipase from *Malbranchea cinnamea*. Biotechnol. Appl. Biochem..

[27] Sun Q, Wang H, Zhang H, Luo H, Shi P, Bai Y, Huang H (2016). Heterologous production of an acidic thermostable lipase with broad-range pH activity from thermophilic fungus *Neosartorya fischeri* P1. J. Biosci. Bioeng..

[28] Zhang X, Li X, Xia L (2015). Expression of a thermo-alkaline lipase gene from *Talaromyces thermophilus* in recombinant Pichiapastoris. Biochem. Eng. J..

[29] Kolling JB, Bertoldo FCA, Brod J, Vernal H, Terenzi ACM, Arisi ACM (2010). Biochemical and structural characterization of two site-directed mutants of *Staphylococcus xylosus* lipase. Mol. Biotechnol..

[30] Li G, Fang X, Su F, Chen Y, Xu L, Yan Y (2018). Enhancing the thermostability of rhizomucor miehei lipase with a limited screening library by rational-design point mutations and disulfide bonds. Appl. Environ. Microbiol..

[31] Stránský K, Zarevúcka M, Kejík Z, Wimmer Z, Macková M, Demneroná K (2007). Substrate specificity, regioselectivity and hydrolytic activity of lipases activated from Geotrichum sp.. Biochem. Eng. J..

[32] Lang DA, Mannesse MLM, De Hass GH, Verheij HM, Dijkstra BW (1998). Structural basis of the chiral selectivity of *Pseudomonas cepacia* lipase. Eur. J. Biochem..

