# Supplementary material for: Characterization of a novel sn1,3 lipase from Ricinus communis L. suitable for production of oleic acid-palmitic acid-glycerol oleate
Source: Sci Rep. 2021 Mar 25;11:6913. doi: 10.1038/s41598-021-86305-z (PMC7994567; doi:10.1038/s41598-021-86305-z)
Supplement: Supplementary file 1 — Supplementary Information. [file 41598_2021_86305_MOESM1_ESM.doc]

**Characterization of a novel sn1,3 lipase from *Ricinus communis* L. suitable for production of oleic acid-palmitic acid-glycerol oleate**

Yue Li^a^, Guorui Li^bcde^, Huajun Sun^f^, Yongsheng Chen^abcde^*


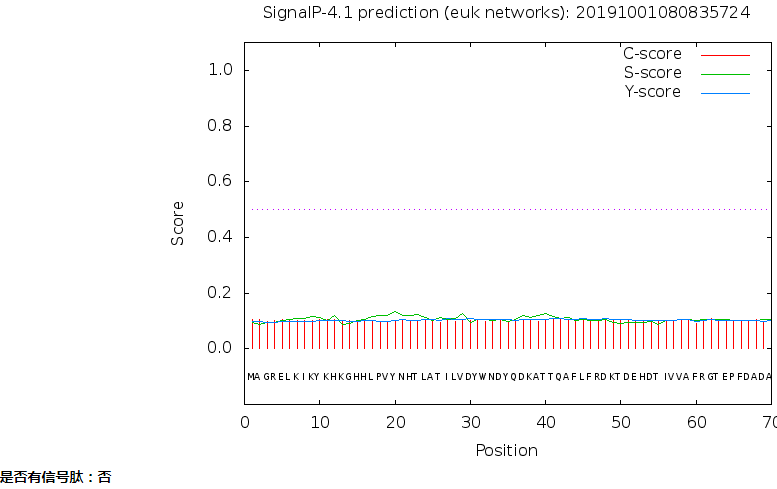


**Figure S1. The prediction of signal peptide of RcLipase.**


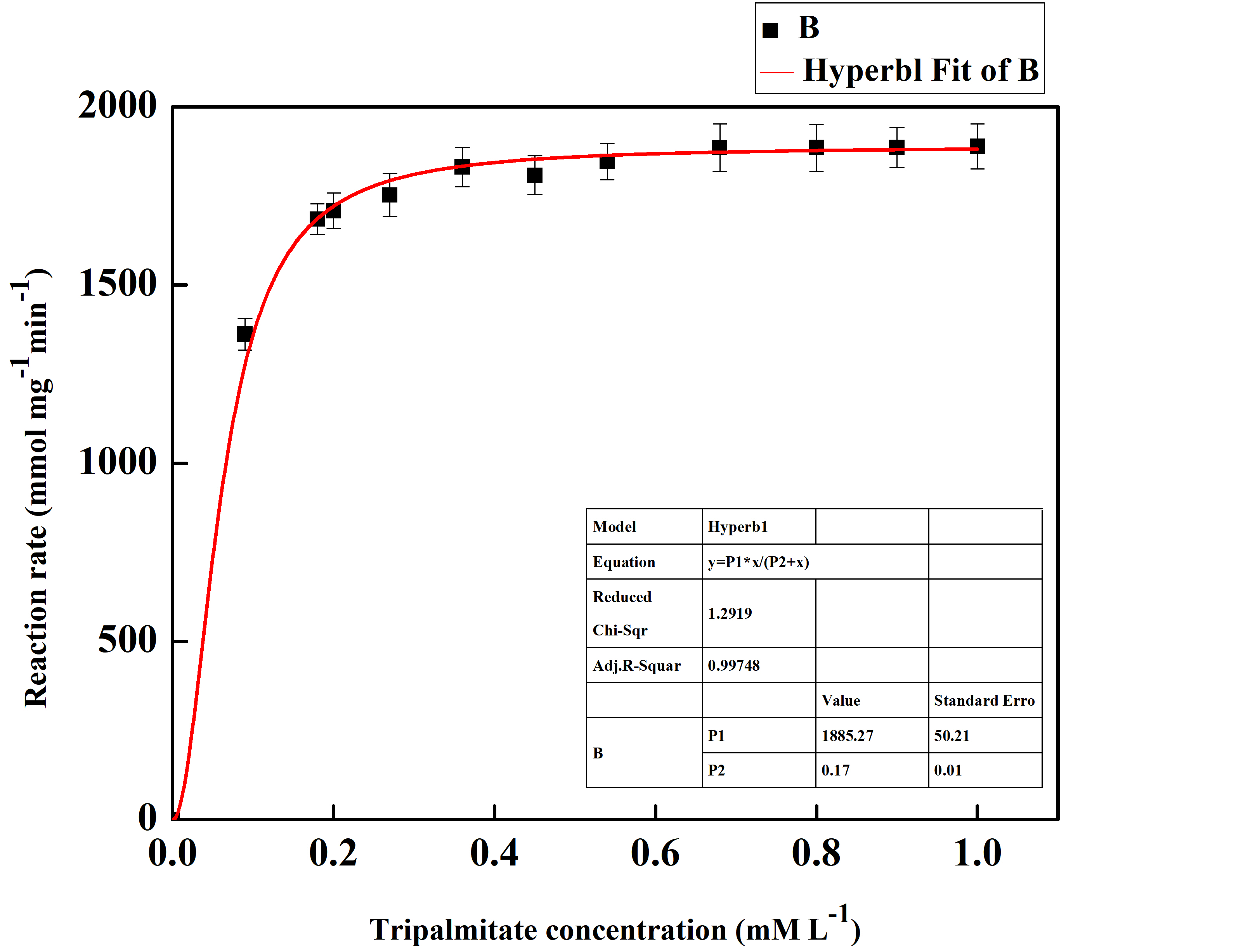


A

**
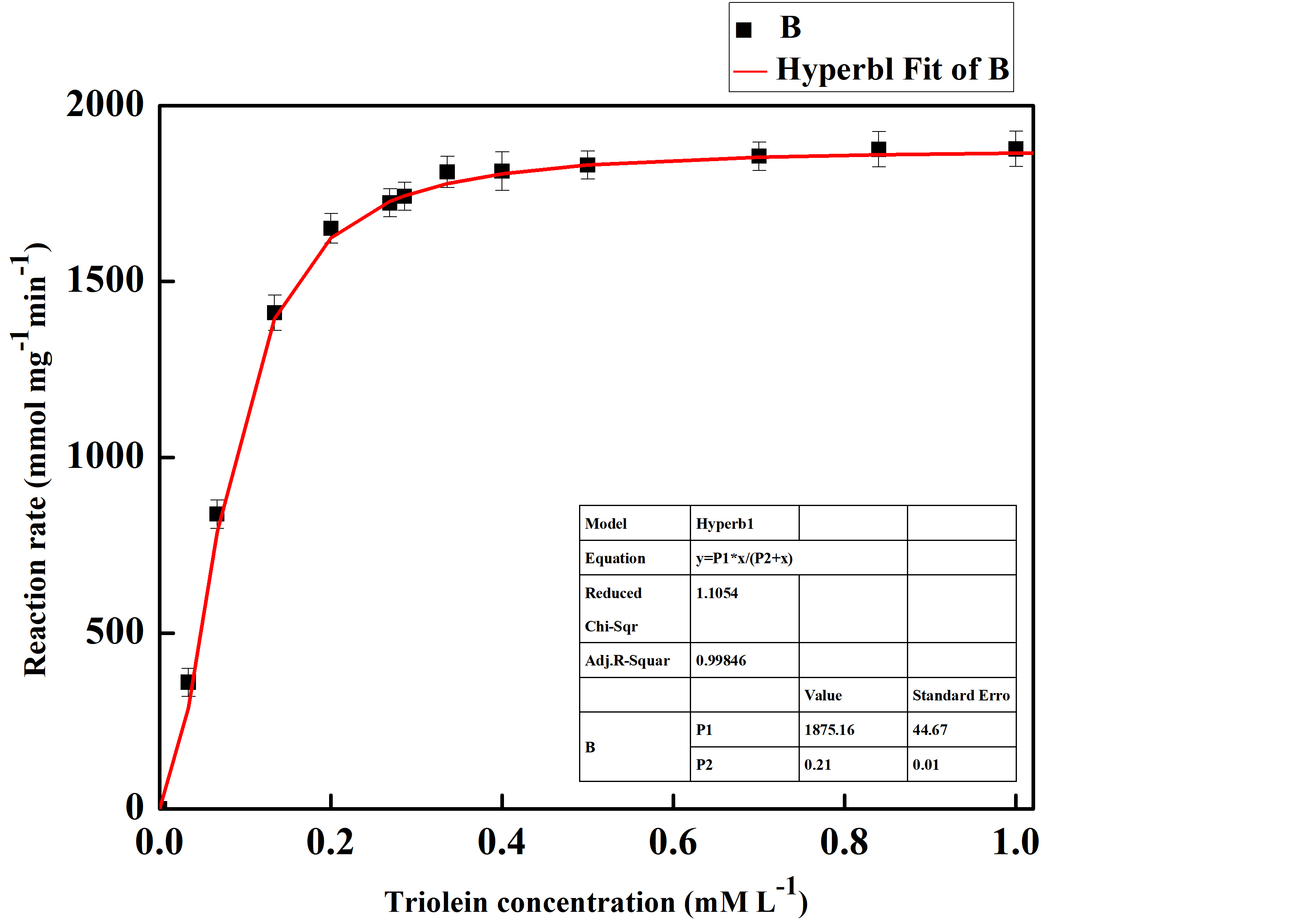
**

B

**
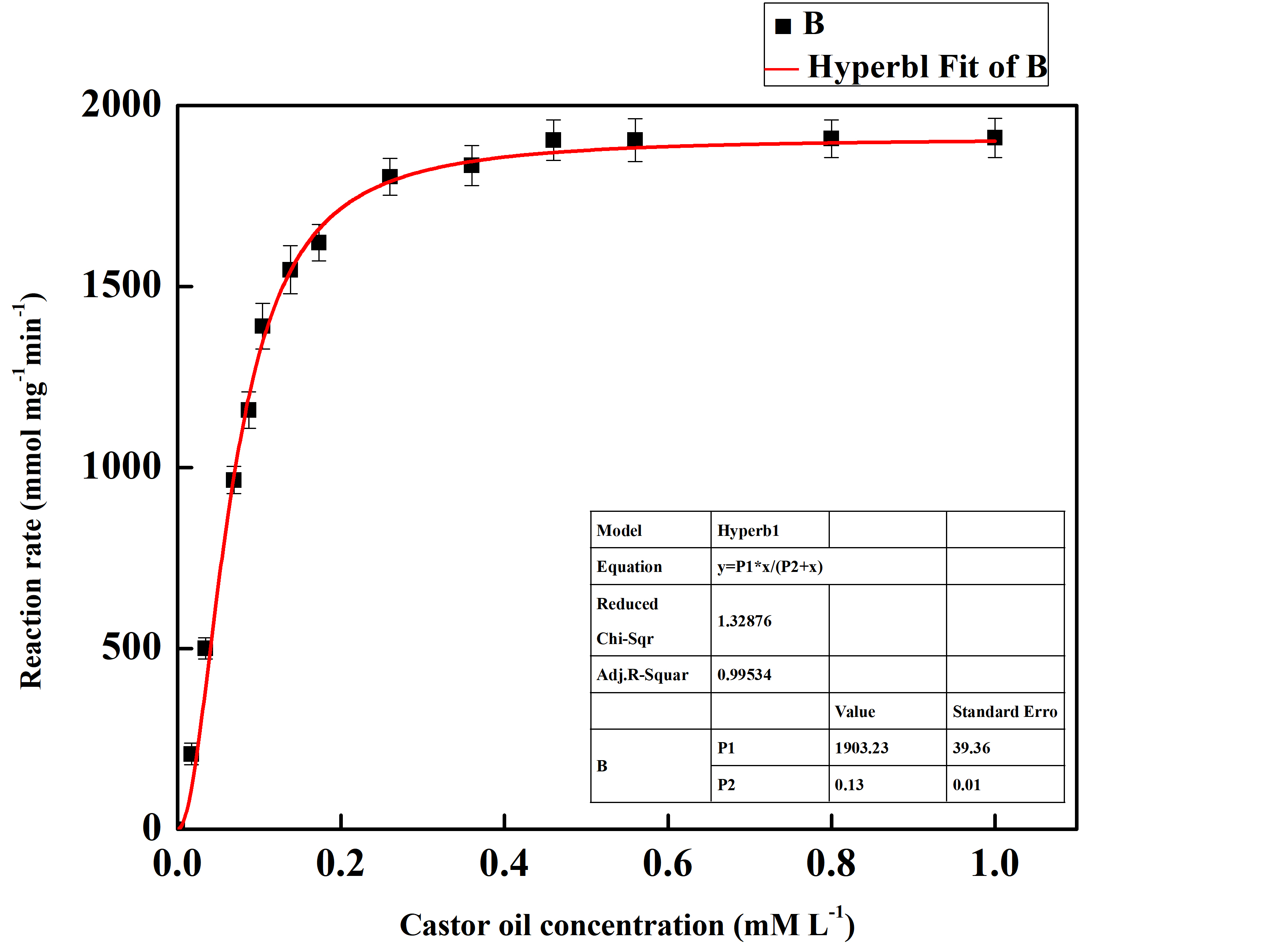
**

C

**Figure S2. Non-linear regression curve analysis of RcLipase.** A. Non-linear regression curve with tripalmitate as substrate; B. Non-linear regression curve with triolein as substrate; C. Non-linear regression curve with castor oil as substrate. The red line represents the fitted curve, and the black dot (B) represents the reaction rate of RcLipase at different substrate concentrations. P1 = *V*_max_ = Value ± Standard Error. P2 = *K*_m_ = Value ± Standard Error.
